# pH-induced change in cell susceptibility to butanol in a high butanol-tolerant bacterium, *Enterococcus faecalis* strain CM4A

**DOI:** 10.1186/s13068-015-0251-x

**Published:** 2015-04-17

**Authors:** Manabu Kanno, Hideyuki Tamaki, Yasuo Mitani, Nobutada Kimura, Satoshi Hanada, Yoichi Kamagata

**Affiliations:** Bioproduction Research Institute, National Institute of Advanced Industrial Science and Technology (AIST), Tsukuba, Ibaraki 305-8566 Japan

**Keywords:** Butanol- and alkali-tolerant microorganism, Physiological response, Butanol and pH stresses, Biofuel

## Abstract

**Background:**

Though butanol is considered as a potential biofuel, its toxicity toward microorganisms is the main bottleneck for the biological butanol production. Recently, butanol-tolerant bacteria have been proposed as alternative butanol production hosts overcoming the end product inhibition. One remaining key issue to be addressed is how physicochemical properties such as pH and temperature affect microbial butanol tolerance during cultivation and fermentation.

**Results:**

We investigated the pH effect on butanol tolerance of a high butanol-tolerant bacterium, *Enterococcus faecalis* strain CM4A. The strain grew over a broad pH range (pH 4.0 to 12.0) and preferred alkaline pH (pH 8.0 and 10.0) in the absence of butanol. However, in the presence of butanol, strain CM4A grew better under acidic and neutral pH conditions (pH 6.0 and 6.8). Membrane fatty acid analysis revealed that the cells exposed to butanol exhibited increased cyclopropane and saturated fatty acids, which contribute to butanol tolerance of the strain by decreasing membrane fluidity, more evidently at acidic and neutral pH than at alkaline pH. Meanwhile, the strain grown under alkaline pH without butanol increased short chain fatty acids, which is involved in increasing membrane fluidity for alkaline adaptation. Such a change was not observed in the cells grown under alkaline pH with butanol. These results suggested that strain CM4A simultaneously exposed to butanol and alkali stresses was not likely able to properly adjust membrane fluidity due to the opposite response to each stress and thereby showed low butanol tolerance under alkaline pH. Indeed, the cells exposed to butanol at alkaline pH showed an irregular shape with disrupted membrane structure under transmission electron microscopy observation, which also indicated the impact of butanol and alkali stresses on functioning of cellular membrane.

**Conclusion:**

The study clearly demonstrated the alkaline pH-induced increase of cell susceptibility to butanol in the tested strain. Our findings indicate the non-negligible impact of pH on microbial butanol tolerance, providing a new insight into efficient butanol production.

**Electronic supplementary material:**

The online version of this article (doi:10.1186/s13068-015-0251-x) contains supplementary material, which is available to authorized users.

## Background

Microbial fuel production from renewable resources has gained increased attention in view of energy security and environmental concerns. Although *n*-butanol (referred to as butanol) is considered to be a promising biofuel due to its desirable chemical properties such as low volatility and miscibility with gasoline [1], butanol fermentation process is still economically unfavorable. Major limitation in biological production of butanol is its high toxicity toward microorganisms [2]. Butanol is known to intercalate into the cell membrane and break the lipid hydrogen bonds, resulting in disruption of membrane structure and cell death [3].

Heterologous butanol production has become realized by the development of genetic and metabolic engineering [4,5], and thus, butanol-tolerant bacteria have been regarded as alternative hosts overcoming growth interruption problem during butanol production. Recently, a number of butanol-tolerant bacteria able to grow in the presence of greater than 2.0% (vol/vol) butanol have been reported [6-11]. We isolated an aerobic butanol-tolerant strain CM4A from grease-contaminated soil, which was most closely related to *Enterococcus faecalis* with high 16S rRNA gene sequence similarity (99.6%) [11]. Strain CM4A exhibited superior butanol tolerance with the ability to grow in the presence of up to 3.5% butanol without assimilation and degradation.

Microbial butanol tolerance is possibly influenced by physicochemical properties such as temperature and pH for cultivation and fermentation. However, very little is known about the influence of those culture conditions to butanol tolerance, since the well-studied bacterial species grew under the limited temperature and pH conditions. Indeed, the effect of culture pH on the butanol tolerance has not been investigated, although only a few studies on the effect of temperature were reported [6,12].

In this study, we aimed to investigate how culture pH affects microbial butanol tolerance using *E. faecalis* strain CM4A, which can grow over a broad pH range (pH 4.0 to 12.0). This study further characterized the response of cell membrane to both butanol and pH stresses.

## Results and discussion

### Alkali tolerance of *E. faecalis* strain CM4A

**Figure 1:**
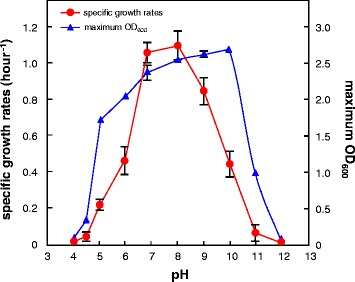
Growth characteristics of *E. faecalis* strain CM4A at various pHs. Effect of pH on growth as determined by specific growth rates (red circle) and maximum optical density at 600 nm (OD_600_) (blue triangle) in the absence of butanol. The values and error bars represent the mean and SD of triplicate experiments.

*E. faecalis* strain CM4A was cultured aerobically in a glucose-rich medium. The strain grew over a broad pH range (pH 4.0 to 12.0), with the highest growth rate at pH 8.0, but the highest maximum optical density at 600 nm (OD_600_) was obtained at pH 10.0 (Figure 1). The alkali tolerance of this strain was comparable to that of obligate alkaliphiles, such as *Alkaliphilus transvaalensis* and *Bacillus marmarensis* [13,14]. Strain CM4A is thus considered as an alkali-tolerant microorganism. The ability to withstand alkaline pH up to 11.9 has been reported in the type strain of *E. faecalis* [15], which is consistent with the results of strain CM4A.

### pH effect on butanol tolerance of *E. faecalis* strain CM4A

**Figure 2:**
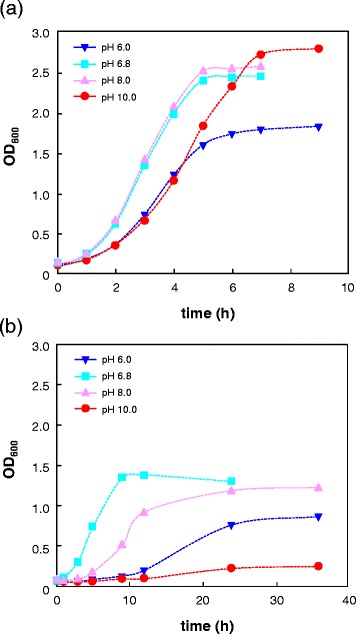
Butanol tolerance assay of *E. faecalis* strain CM4A. Growth curves of strain CM4A in the absence **(a)** and presence **(b)** of 2.0% butanol under different pH conditions. The values represent the mean of triplicate experiments. OD_600_, optical density at 600 nm.

To elucidate an effect of pH on butanol tolerance, strain CM4A was tested for butanol tolerance under various pHs. Since cells did not grow sufficiently at pH 5.0 or pH 11.0 in the presence of 2.0% butanol (data not shown), the relative growth rates were compared under four different pH conditions (pH 6.0, 6.8, 8.0, 10.0) in the presence and absence of butanol. Interestingly, the strain exhibited the highest growth rate at neutral pH (pH 6.8) in the presence of 2.0% butanol, although its optimum growth was observed at alkaline pH (pH 8.0) in the absence of butanol (Figure 2). Note that this alkali-tolerant strain also showed significantly higher growth rate at pH 6.0 than at pH 10.0 in the presence of butanol (p < 0.01 by *t*-test). Likewise, other butanol-tolerant strains, *Bacillus amyloliquefaciens* strain FW5A and *Lysinibacillus xylanilyticus* strain SK7A in the phylum *Firmicutes* [11], also showed relatively high butanol tolerance at acidic pH conditions (see Additional file 1 and Additional file 2), suggesting that this phenomenon might be a common trait in butanol-tolerant strains within the *Firmicutes*.

### Changes in the fatty acid compositions of *E. faecalis* strain CM4A in the absence of butanol at alkaline pH

To elucidate the mechanism of alkali tolerance, the response of the cell membrane to alkali challenge was further investigated. In particular, changes in the fatty acid composition of strain CM4A were determined under different pH conditions (pH 6.0, 6.8, 8.0, 10.0) in the absence of butanol. The cellular fatty acids of strain CM4A were mainly composed of cyclopropane and normal saturated or unsaturated chains in the range from C14 to C20, with a high abundance of palmitic acid (C16:0) and *cis*-vaccenic acid (C18:1ω7c). No significant change was observed between acidic and neutral pH (p > 0.01 by *t*-test). In contrast, the proportion of C14 chain length fatty acids (myristic acid (C14:0) and *cis*-tetradecenoic acid (C14:1ω7c)) tended to increase with increasing pH (Table 1). Such increase in the proportion of short chain fatty acids are known to increase membrane fluidity [16] that is a key to microbial alkaline adaptation. It has been reported, indeed, that *E. coli*, *Listeria monocytogenes*, and alkaliphilic *Bacillus* spp. also enhance membrane fluidity under alkaline conditions by increasing unsaturated or branched-chain fatty acids [17-20]. Membrane fluidity is known to affect the configuration and activity of membrane proteins such as ATP synthase and various transporters (for example, cation/proton antiporters, and so on), which are regulating proton entry and cytoplasmic retention that serve to prevent intracellular alkalinization [21-23]. Taken together, our findings revealed that strain CM4A altered membrane fatty acid compositions for increasing its membrane fluidity and subsequently adapted to alkaline conditions.

**Table 1:** Changes in the fatty acid composition of strain CM4A*

	**No solvents**				**2.0% butanol**			
	**pH 6.0**	**pH 6.8**	**pH 8.0**	**pH 10.0**	**pH 6.0**	**pH 6.8**	**pH 8.0**	**pH 10.0**
C14:0	4.2 ± 0.5	4.6 ± 1.3	6.5 ± 1.9	6.9 ± 0.7	5.8 ± 0.3	5.0 ± 1.9	5.8 ± 0.1	4.6 ± 1.9
C14:1ω7c	0.1 ± 0.2	0.2 ± 0.2	0.5 ± 0.2	0.6 ± 0.1	0.3 ± 0.3	0.3 ± 0.3	0.6 ± 0.1	0.2 ± 0.2
C16:0	41.5 ± 1.1	41.6 ± 1.0	39.6 ± 3.3	39.2 ± 2.9	49.4 ± 4.1	44.8 ± 1.8	41.0 ± 1.9	43.5 ± 1.7
C16:1	0.8 ± 0.7	1.2 ± 1.1	0.9 ± 0.9	0.9 ± 0.9	1.0 ± 1.0	1.9 ± 2.0	1.3 ± 1.5	0.8 ± 0.7
C16:1ω7c	4.4 ± 1.3	6.1 ± 2.0	6.5 ± 0.8	7.5 ± 1.1	6.4 ± 1.2	7.2 ± 2.6	7.5 ± 1.8	6.9 ± 4.4
C18:0	2.2 ± 0.6	1.5 ± 0.2	1.3 ± 0.5	0.9 ± 0.2	2.7 ± 1.2	1.6 ± 0.6	1.2 ± 0.6	5.1 ± 3.6
C18:1ω7c	46.3 ± 2.4	44.9 ± 4.8	44.3 ± 3.0	43.8 ± 1.7	30.4 ± 0.9	37.6 ± 4.9	42.0 ± 4.1	38.6 ± 2.8
cyclo-C19:0	0.6 ± 0.8	ND	0.4 ± 0.7	ND	*3.5 ± 1.1*	*1.3 ± 0.6*	*0.7 ± 0.2*	*0.3 ± 0.3*
C20:1	0.0 ± 0.1	ND	0.1 ± 0.1	ND	0.5 ± 0.9	0.1 ± 0.2	ND	ND
Total C14	*4.3 ± 0.5*	*4.8 ± 1.5*	*6.9 ± 2.1*	*7.5 ± 0.8*	*6.1 ± 0.6*	*5.4 ± 2.3*	*6.4 ± 0.1*	*4.8 ± 1.8*
Total saturated fatty acids	47.9 ± 0.6	47.6 ± 1.6	47.4 ± 4.7	47.1 ± 3.3	*57.9 ± 4.7*	*51.5 ± 1.6*	*47.9 ± 1.4*	*53.2 ± 1.8*

*Each fatty acid composition is described as a percentage of the whole-cell lipids. The values are the means ± standard deviations of three independent measurements. The double-bond positions of C16:1 and C20:1 were not identified. ‘Total saturated fatty acids’ is the sum of myristic acid (C14:0), palmitic acid (C16:0), and stearic acid (C18:0). Abbreviations: C14:1ω7c, *cis*-tetradecenoic acid; C16:1ω7c, palmitoleic acid; C18:1ω7c, *cis*-vaccenic acid; cyclo-C19:0, *cis*-11,12-methylene octadecanoic acid; ND, not detected. The italicized values are discussed in the text.

### Changes in the fatty acid compositions of *E. faecalis* strain CM4A in the presence of butanol at different pHs

The fatty acid composition of strain CM4A was also investigated at different pHs in the presence of 2% butanol. The increase in the proportion of both cyclopropane fatty acid (*cis*-11,12-methylene octadecanoic acid (cyclo-C19:0)) and total saturated fatty acids in response to butanol was found regardless of the culture pH (Table 1). These alterations causing a decrease in membrane fluidity likely contribute to maintain cellular integrity upon exposure to butanol as suggested in the previous studies [3,11,24]. It is noteworthy that the responses of the cell membrane to butanol exposure were more evident at acidic pH (Table 1). For example, the cells grown at pH 6.0 contained higher proportions of cyclopropane fatty acid (3.5 ± 1.1%) and total saturated fatty acids (57.9 ± 4.7%) than the cells grown at other pH conditions. In contrast, these changes were little observed in the cells grown at pH 8.0 and 10.0, although the proportion of saturated fatty acids was slightly high at pH 10.0 (53.2 ± 1.8%). The results suggested that the cells failed to decrease membrane fluidity at alkaline pHs in the presence of butanol, which appeared to be a main cause of lowered butanol tolerance under alkaline conditions.

**Figure 3:**
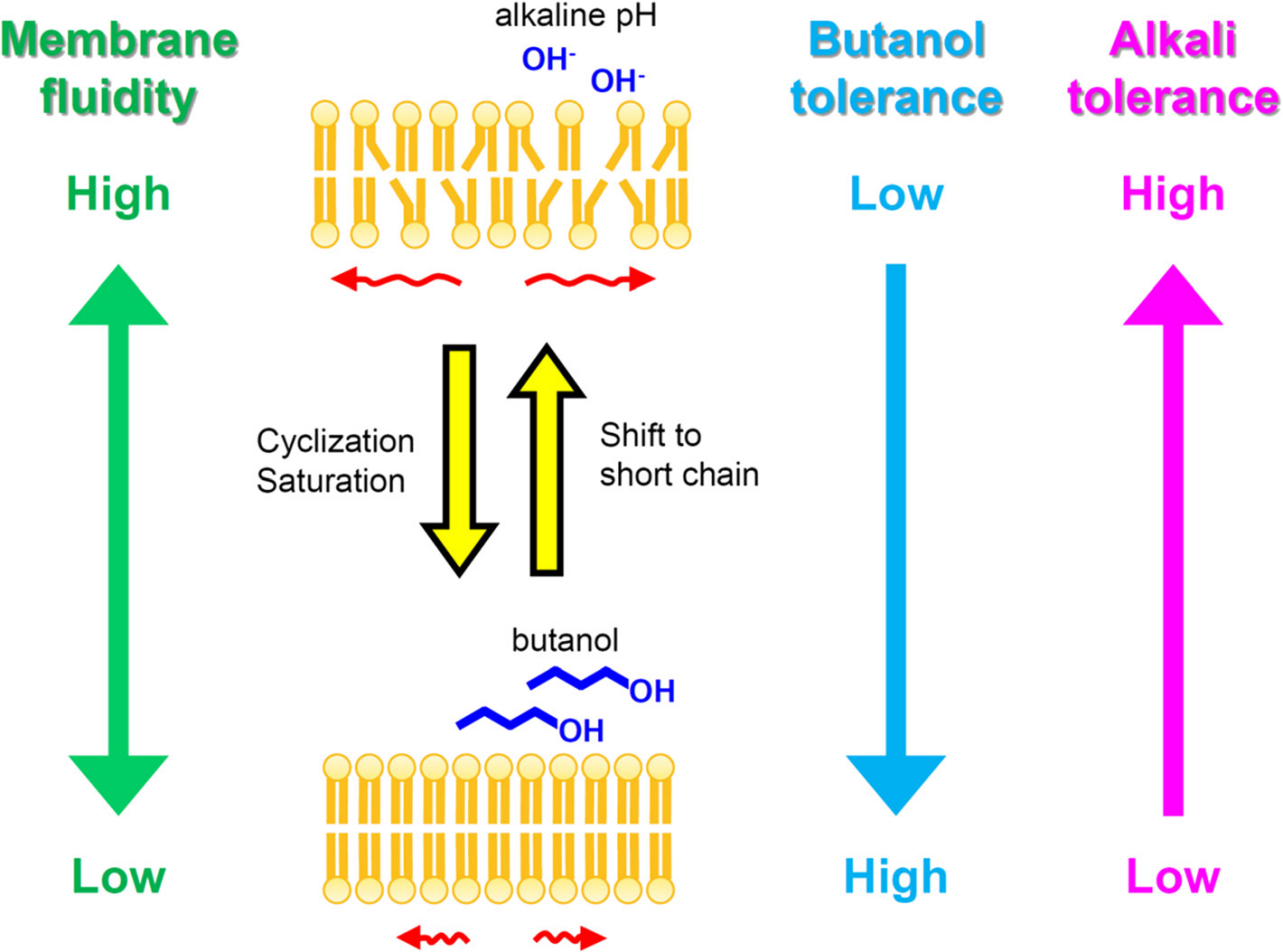
Schematic representation of membrane fluidity adjustment in *E. faecalis* strain CM4A. The opposite mechanisms involved in membrane fluidity adjustment behind butanol and alkali tolerance. The strain alters the proportion of membrane fatty acid components (that is cyclization, saturation, or shift to short chain) in response to butanol exposure or alkaline pH.

**Figure 4:**
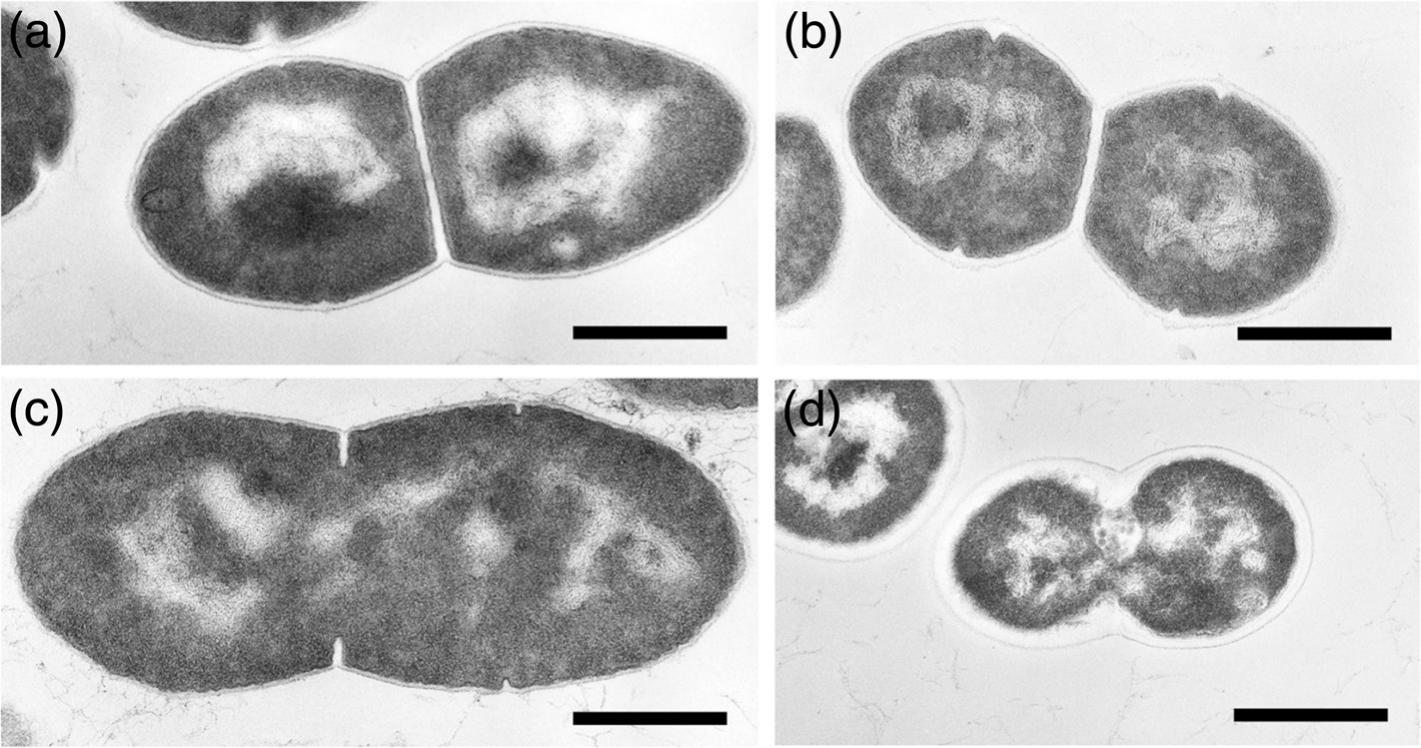
Cell morphology of *E. faecalis* strain CM4A. Transmission electron micrographs of the cell grown at pH 6.8 **(a, b)** or pH 10.0 **(c, d)** in the absence **(a, c)** and presence **(b, d)** of 2.0% butanol. Bars, 0.5 μm.

Furthermore, the increase in the proportion of C14 chain length fatty acids with increasing pH, which occurred in the absence of butanol, was not observed in the cells grown with butanol (Table 1). This indicated that the adjustment of membrane fluidity in response to alkali challenge did not work in the presence of butanol. Thus, whereas strain CM4A would tolerate butanol by decreasing membrane fluidity, it would adapt to alkaline pH by increasing membrane fluidity (Figure 3). Due to these two mechanisms working oppositely, the strain might lose the ability to adjust membrane fluidity under simultaneous exposure to both butanol and alkali stresses; hence, the strain might increase cell susceptibility to butanol under alkaline conditions. Indeed, by transmission electron microscopy, the cells grown with 2.0% butanol at pH 10.0 showed irregular shape with disrupted membrane structure (Figure 4). This is contrary to the cells grown in other conditions, which showed well-preserved cell structure (Figure 4).

## Conclusions

In this paper, we present a conceptual study of combined effects of butanol and pH stresses on bacterial cells. It is convincingly concluded that a high butanol-tolerant bacterium, *E. faecalis* strain CM4A, shows lower ability to tolerate butanol under alkaline conditions due to the interactive effects of butanol and alkali stresses. To our knowledge, this is the first report describing the effect of pH on microbial butanol tolerance. High butanol-tolerant bacteria can be considered as an alternative butanol production host. Our findings indicate the non-negligible impact of pH on butanol tolerance of bacterial species, which provide a new insight into an efficient heterologous butanol production using alternative bacterial host.

## Methods

### Strains and culture conditions

Previously isolated butanol-tolerant strains able to grow in greater than 2.0% (vol/vol) butanol at pH 6.8, *E. faecalis* CM4A, *Bacillus* sp. FW5A, and *Lysinibacillus* sp. SK7A in the phylum *Firmicutes* [11], were used in this study. Cells were grown aerobically with shaking at 30°C in tryptone-glucose-yeast (TGY) medium consisting of (per liter) 20 g tryptone, 5 g glucose, 5 g yeast extract, 7 ml basal salt solution, 1 ml vitamin solution [25], and 25 mM buffer as described below.

### Growth test under different pH conditions

To assess the effect of pH, strains were cultured under various pH conditions (pH 3.0 to 12.5). Growth was monitored by measuring the optical density (OD_600_) as previously described [6,8-11]. The pH value of culture medium was adjusted by using the following appropriate buffer: sodium citrate buffer (pH 3.0), sodium acetate buffer (pH 4.0 to 5.0), potassium phosphate buffer (pH 6.0 to 9.0), sodium carbonate buffer (pH 10.0), and disodium hydrogen phosphate buffer plus NaOH (pH 11.0 to 12.5). After autoclaving, the pH value was measured by a pH meter and readjusted by adding NaOH or HCl. The pH value did not change by more than 0.3 from the initial pH during the period of exponential growth, in which physiological and morphological characterization was conducted.

### Butanol tolerance assay

Growth in the presence of *n*-butanol was evaluated in the same way as mentioned above. Specific growth rate was calculated from the linear range of exponential growth. Relative growth rate was defined as the specific growth rate in the presence of butanol relative to that without butanol.

### Physiological and morphological characterization

Membrane fatty acid composition and cell morphology of strain CM4A in the late exponential growth phase were investigated as previously described [11]. Briefly, fatty acid methyl esters were extracted from whole-cell methanolyzed products into *n*-hexane and then analyzed by gas chromatography-mass spectrometry system. Cell morphology was observed by using transmission electron microscopy as previously described [26].

## Additional files

Additional file 1: Figure S1. Butanol tolerance assay of *Bacillus amyloliquefaciens* strain FW5A. Growth curves of strain FW5A in the absence (a) and presence (b) of 2.0% butanol under different pH conditions. The values represent the mean of triplicate experiments. Additional file 2: Figure S2. Butanol tolerance assay of *Lysinibacillus xylanilyticus* strain SK7A. Growth curves of strain SK7A in the absence (a) and presence (b) of 2.0% butanol under different pH conditions. The values represent the mean of triplicate experiments.

## Abbreviations

C14:0: myristic acid
C14:1ω7c: *cis*-tetradecenoic acid
C16:0: palmitic acid
C16:1ω7c: palmitoleic acid
C18:0: stearic acid
C18:1ω7c: *cis*-vaccenic acid
cyclo-C19:0: *cis*-11,12-methylene octadecanoic acid
OD_600_: optical density at 600 nm
TGY: tryptone-glucose-yeast
