# Supplementary figures and images for: pH-induced change in cell susceptibility to butanol in a high butanol-tolerant bacterium, Enterococcus faecalis strain CM4A
Source: Biotechnol Biofuels. 2015 Apr 17;8:69. doi: 10.1186/s13068-015-0251-x (PMC4405824; doi:10.1186/s13068-015-0251-x)

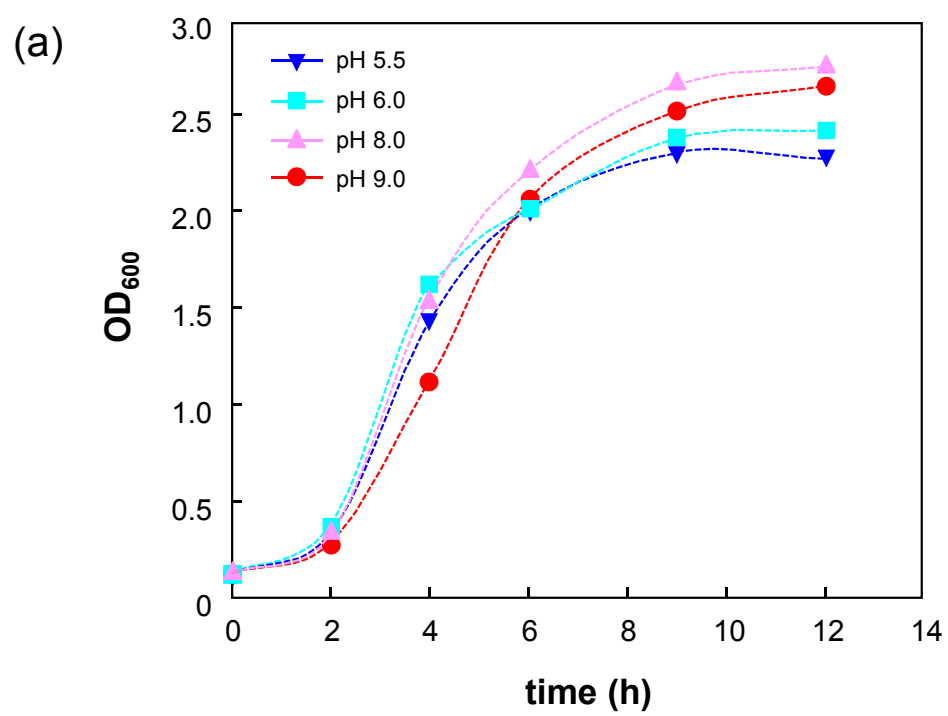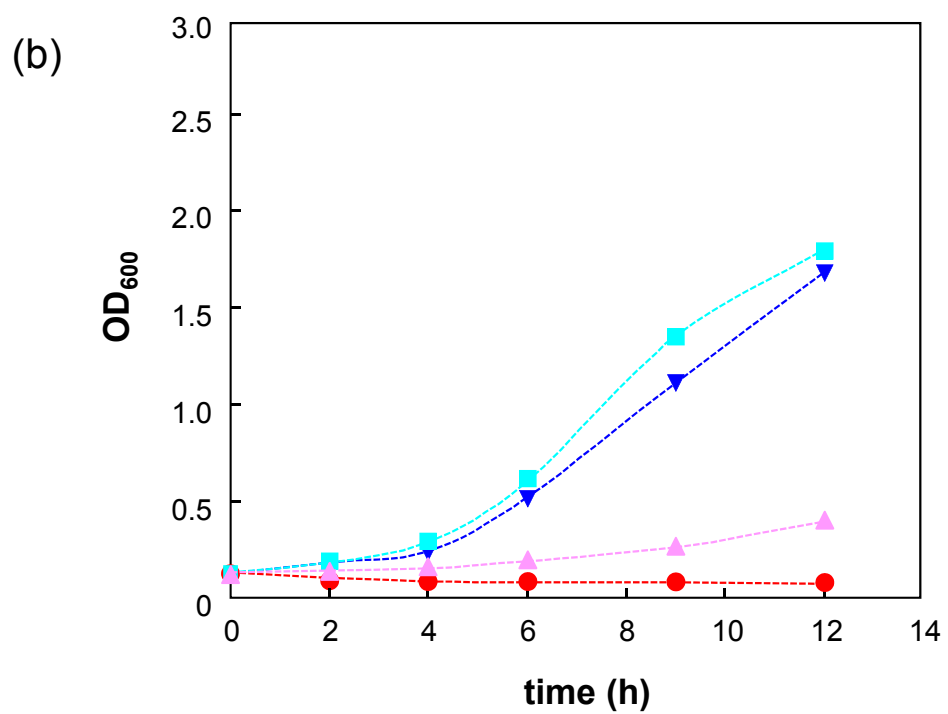

Supplement: Additional file 1: Figure S1. — Butanol tolerance assay of Bacillus amyloliquefaciens strain FW5A. Growth curves of strain FW5A in the absence (a) and presence (b) of 2.0% butanol under different pH conditions. The values represent the mean of triplicate experiments. [file 13068_2015_251_MOESM1_ESM.pdf]

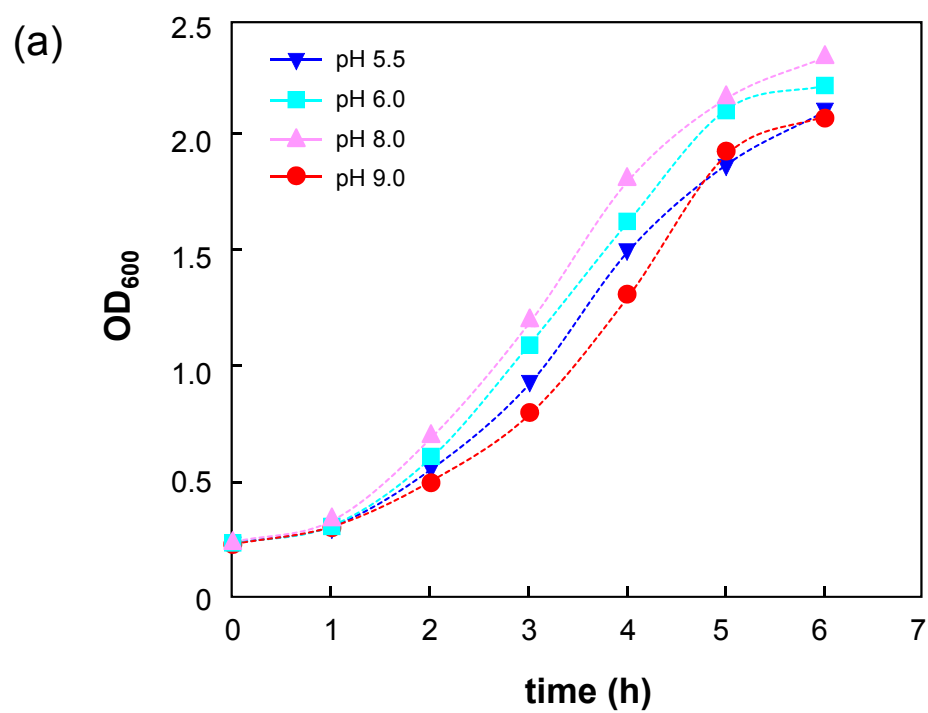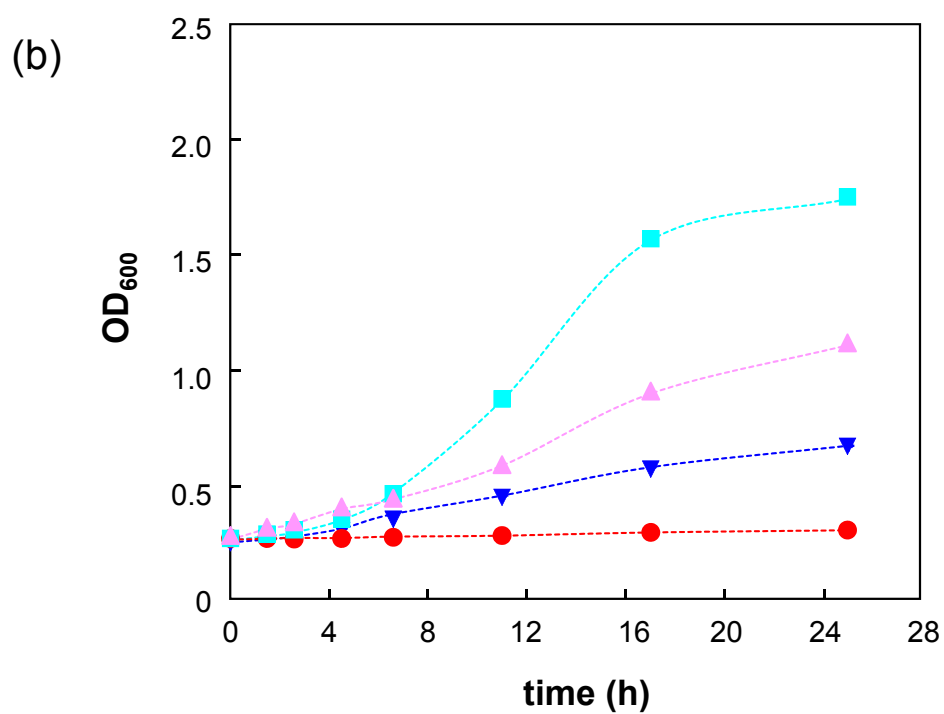

Supplement: Additional file 2: Figure S2. — Butanol tolerance assay of Lysinibacillus xylanilyticus strain SK7A. Growth curves of strain SK7A in the absence (a) and presence (b) of 2.0% butanol under different pH conditions. The values represent the mean of triplicate experiments. [file 13068_2015_251_MOESM2_ESM.pdf]
